# Brain morphometric changes in children born as small for gestational age without catch up growth

**DOI:** 10.3389/fnins.2024.1441563

**Published:** 2024-08-29

**Authors:** Tomozumi Takatani, Tadashi Shiohama, Rieko Takatani, Shinya Hattori, Hajime Yokota, Hiromichi Hamada

**Affiliations:** ^1^Department of Pediatrics, Graduate School of Medicine, Chiba University, Chiba, Japan; ^2^Faculty of Education, Graduate School of Education, Chiba University, Chiba, Japan; ^3^Department of Radiology, Chiba University Hospital, Chiba, Japan; ^4^Department of Diagnostic Radiology and Radiation Oncology, Graduate School of Medicine, Chiba University, Chiba, Japan

**Keywords:** idiopathic short stature, short for gestational age, voxel-based morphometry, surface-based morphometry, brain magnetic resonance imaging

## Abstract

**Introduction:**

Most infants born as small for gestational age (SGA) demonstrate catch up growth by 2–4 years, but some fail to do so. This failure is associated with several health risks, including neuropsychological development issues. However, data on the morphological characteristics of the brains of infants born as SGA without achieving catch up growth are lacking. This study aims to determine the structural aspects of the brains of children born as SGA without catch up growth.

**Methods:**

We conducted voxel- and surface-based morphometric analyses of 1.5-T T1-weighted brain images scanned from eight infants born as SGA who could not achieve catch up growth by 3 years and sixteen individuals with idiopathic short stature (ISS) to exclude body size effects. Growth hormone (GH) secretion stimulation tests were used to rule out GH deficiency in all SGA and ISS cases. The magnetic resonance imaging data were assessed using Levene’s test for equality of variances and a two-tailed unpaired *t*-test for equality of means. The Benjamini–Hochberg procedure was used to apply discovery rate correction for multiple comparisons.

**Results:**

Morphometric analyses of both *t*-statical map and surface-based analyses using general linear multiple analysis determined decreased left insula thickness and volume in SGA without catch up growth compared with ISS.

**Conclusion:**

The brain scans of patients with SGA who lack catch up growth indicated distinct morphological disparities when compared to those with ISS. The discernible features of brain morphology observed in patients born as SGA without catch up growth may improve understanding of the association of SGA without catch up growth with both intellectual and psychological outcomes.

## 1 Introduction

Global efforts in public health focus on advancing maternal and child health to decrease low birth weight incidence [World Health Organization Global Nutrition Targets 2025: Low Birth Weight Policy Brief: WHO, 2014. 2014 (accessed on December 7, 2023) Available online]. Fetal growth restriction is a primary contributor to low birth weight that increases the likelihood of mortality and morbidity in infants (Bernstein et al., 2000; Malhotra et al., 2019). Additionally, it presents a notable risk of developmental delay and impaired cognitive function in later childhood (Levine et al., 2015; Rogne et al., 2015).

Infants who are classified as small for gestational age (SGA) are those whose birth weight falls below the tenth percentile. Changes in fetal growth standards and risk factors have increased the detection rate and the number of infants born as SGA. Approximately 85–90% of infants born as SGA undergo spontaneous catch up growth within the first 2 years after birth, facilitated by environmental influences (Karlberg and Albertsson-Wikland, 1995; Campisi et al., 2019). However, some children cannot catch-up for 2–4 years (Gibson et al., 2000; Zanelli and Rogol, 2018). This failure is associated with neurodevelopmental function. One report revealed the failure of catch up growth as a strong predictor for subnormal intellectual and psychological performance (Lundgren et al., 2001). Another study demonstrated that the failure of catch up growth is associated with impaired neurobehavioral development, with an increased risk for aggressive behaviors (Takeuchi et al., 2018). These studies indicate an association between the absence of catch up growth and impaired brain function. Understanding the causal relationships and mechanisms in children born as SGA without catch up growth is crucial, considering the significant impact on their future psychological well-being. However, specific changes that explain this association remain unclear. Recently, the focus on detailed investigations of changes in brain morphology has increased to improve our understanding of neurological disorders (Shiohama et al., 2019a,b, 2020).

Quantitative analysis revealed that one report demonstrated lower regional brain volume in superior and anterior brain areas in children born as SGA without catch up growth (Tzarouchi et al., 2014). However, the comparison with normal height children and SGA with catch up growth is an approach that does not eliminate the influence of the short stature itself, and the association of the inability to catch-up with brain morphology is unknown. Moreover, we now achieved more detailed brain morphology data, including cortical thickness, due to technological advances. Hence, we conducted a study comparing the brain morphology of children born as SGA without catch up growth with that of children who demonstrated short stature without a defined cause, such as growth hormone deficiency (GHD) or Turner syndrome, also known as idiopathic short stature (ISS). It helps to minimize the height effect on brain morphology. Our study aimed to characterize the structural aspects of the brains of children born as SGA without catch up growth, using region- and surface-based measurements obtained through advanced structural magnetic resonance imaging (MRI) techniques with comprehensive brain MRI measurement.

## 2 Methods

### 2.1 Patients

**Figure 1:**
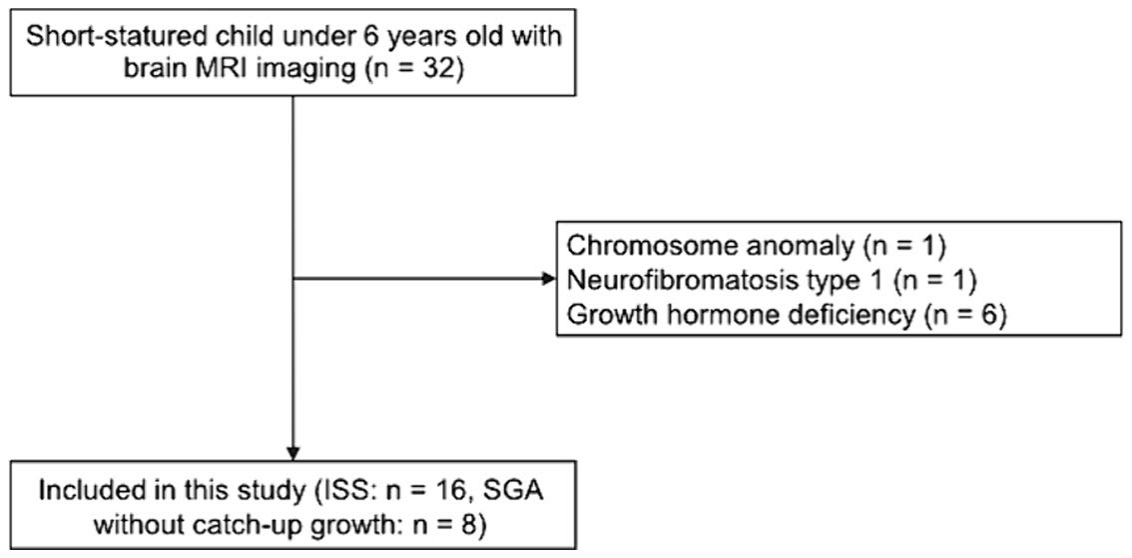
Flow chart of included participants. From the 32 short-statured children under 6 years old with brain MRI images, we excluded those with a chromosome anomaly or neurofibromatosis type 1. Additionally, we excluded those with growth hormone deficiency based on the growth hormone stimulation test. Finally, 24 short-statured children under 6 years old with brain MRI images were analyzed.

We gathered a sample of eight patients born as SGA without catch up growth by 3 years from electronic medical records from 2016 to 2020 at Chiba University Hospital, which is a tertiary medical center. We excluded the clinical diagnoses of SGA without catch up growth for all patients in our sample from the GHD confirmed by a certified pediatric endocrinologist using the clinical course and GH stimulation tests. Additionally, patients with intracranial tumors and other malformations were excluded. ISS was also excluded from the GHD by the GH stimulation test (Figure 1). Moreover, a certified pediatric endocrinologist confirmed that the study participants with short stature do not have endocrine disorders that cause short stature, including hypothyroidism or Turner’s syndrome. We collected the medical records and MRI datasets for all participants. Both the SGA and ISS in MRI datasets were acquired at Chiba University Hospital on the same MRI scanner suite.

### 2.2 Structural MRI acquisition and processing

Three-dimensional (3D) spoiled gradient-echo T1-weighted sagittal images (repetition time/echo time: 7–22/2–5 ms, slice thickness: 1.0–1.4 mm, voxel size: 0.5 × 0.5 × 1 mm, and matrix: 256 × 256) were obtained from all the included participants in this study using clinical 1.5-T MRI scanners (GE Signa HDxT 1.5 T, GE Healthcare, Milwaukee, WI, United States). The CIVET version 2.1.0 pipeline (Zijdenbos et al., 2002) on the Canadian Brain Imaging Research (CBRAIN) platform was used for DICOM file analysis (Sherif et al., 2014). The N3 algorithm (Sled et al., 1998), stereotaxic registration (onto the icbm152 nonlinear 2009 template) (Fonov et al., 2009), and brain masking (Smith, 2002) were used for nonuniform intensity artifact corrections.

Voxel-based volumetric analysis was conducted with tissue classification using an artificial neural network classifier (INSECT) (Tohka et al., 2004) according to icbm152nl_09s (MNI ICBM152 non-linear symmetric 2009), 12 degrees of freedom for linear registration, trilinear interpolation, and 200 mm of N3 distance. Additionally, brain regions were performed with anatomical labeling (ANIMAL) parcellation (Collins et al., 1999) based on the icbm152nl-VI (MNI ICBM152 generation VI symmetric model).

Gray and white matter surfaces for our surface analysis were extracted with 40,962 vertices per hemisphere using the t-Laplace metric (Kim et al., 2005; Boucher et al., 2009) according to icbm152MCsym (MNI ICBM152, marching-cubes, symmetric 2014). They were smoothed over the surface using a full width at half maximum of 8 mm t-Laplace in the cortical thickness metric. Cortical surface parameters, including the gyrification index (GI), average cortex thickness, cortical surface area, and cortical volumes, were calculated for each hemisphere. Desikan–Killiany–Tourville atlas was used for the surface parcellation. The output of the CIVET pipeline (brain mask shapes, linear and nonlinear registration to the template, tissue classification, and brain segmentation) was manually inspected for quality.

### 2.3 Statistical analyses

A two-tailed unpaired Welch’s *t*-test for the non-equality of means and Cohen’s *d* was used to evaluate each structural measurement. Absolute Cohen’s *d* of 0.8 was recognized as the cut-off value for large-size effects. The Benjamini–Hochberg procedure was used for false discovery rate correction for multiple comparisons (Benjamini et al., 2001; Reiner et al., 2003). Benjamini–Hochberg critical values (*q* = 0.15) were determined as *p* of <4.4 × 10^−3^ for 241 repeating *t*-tests for measurements. The General Linear Model (*p* < 0.05) was used to evaluate the effects of continuous (age at scan) or binary (sex) covariates. SPSS v. 28 (IBM Corp. Armonk, NY, United States) software was used for all statistical analyses for each measurement. Regional cortical thicknesses were statistically analyzed and visualized as t-statistical maps and random field theory (RFT) maps using the SurfStat toolbox^1^ with MATLAB R2016a (MathWorks, Natick, MA, United States). The threshold of *p-*value for RFT at cluster level was 0.02.

## 3 Results

### 3.1 Participants’ background

Table 1 shows the relevant characteristics of both the participants with ISS (*N* = 16) and those with SGA without catch up growth (*N* = 8). The current study included no one with brain tumors. Additionally, participants have no intellectual disabilities, autism spectrum disorder, adult attention-deficit/hyperactivity disorder (ADHD), or epilepsy. Study participants in the ISS group underwent MRI scans at slightly but significantly older ages compared to those in the SGA group without catch up growth (mean ± standard deviation [SD] = 4.1 ± 0.7 and 3.4 ± 0.3 years old in ISS and SGA participants, respectively). Brain MRI qualitative analyses revealed no abnormal parenchymal results in either participants with SGA or ISS. The mean ± SD of GH peaks in patients with ISS and SGA without catch up growth, as determined by GH stimulation tests, were 14.0 ± 12.8 and 12.6 ± 8.0 ng/mL, respectively, with no statistically significant difference. Similarly, insulin like growth factor-1 levels did not differ between the two groups. Body composition parameters, such as height and weight, demonstrated no statistically significant differences between the ISS and SGA groups.

**Table 1:** Demographics and clinical characteristics.

Characteristics	ISS (*N* = 16)	SGA (*N* = 8)	*p*-value
Age (years)	4.1 (0.7)	3.4 (0.3)	0.01
Sex			1.00
Male	10	5	
Female	6	3	
Height SDS	−3.0 (0.6)	−3.0 (0.4)	0.89
BMI SDS	−3.2 (1.0)	−0.8 (1.3)	0.29
IGF-1 (ng/ml)	63.6 (26.1)	68.5 (17.3)	0.65
<GH stimulation test>			
GH peak (ng/ml)	14.0 (12.8)	12.6 (8.0)	0.78

Mean (standard deviation), Student’s *t*-test was used to compare the means between the two groups. The chi-square test was utilized to assess the disparity in sex distribution between the two groups.

BMI, body mass index; ISS, idiopathic short stature syndrome; SGA, short for gestational age; GH, growth hormone; IGF-1, insulin like growth factor-1; SDS, standard deviation score.

### 3.2 Global brain measurements

Cortical volume, surface area, cortical thickness, and GIs exhibited no significant differences between the ISS and SGA groups (Table 2).

**Table 2:** The global brain volume and cortical surface measurements in participants born as SGA without catch up growth and those with ISS.

	SGA (*N* = 8) Mean [SD]	ISS (*N* = 16) Mean [SD]	The rate of ISS/SGA	*p*	Absolute Cohen’s *d*
CSF (mm^3^)	35,958 [21958]	36,293 [5751]	0.99	0.967	−0.025
Cortical GM (mm^3^)	553,957 [59335]	597,711 [49751]	0.93	0.098	−0.83
WM (mm^3^)	282,924 [46509]	317,809 [43791]	0.89	0.1	−0.78
Subcortical GM (mm^3^)	29,090 [3672]	32,359 [3937]	0.9	0.063	−0.85
Gyrification Index	3.67 [0.14]	3.69 [0.16]	0.99	0.703	−0.16
L gyrification index	2.65 [0.09]	2.71 [0.11]	0.98	0.165	−0.59
R gyrification index	2.71 [0.11]	2.73 [0.12]	0.99	0.65	−0.19
L cortex average thickness (mm)	3.13 [0.15]	3.13 [0.09]	1	0.905	0.064
R cortex average thickness (mm)	3.17 [0.15]	3.16 [0.08]	1	0.875	0.086
L cortex surface area (mm^2^)	83,062 [6539]	90,227 [6863]	0.92	0.025	−1.06
R cortex surface area (mm^2^)	84,079 [6741]	90,654 [7477]	0.93	0.046	−0.91
L cortex volume (mm^3^)	248,809 [25854]	271,810 [20365]	0.92	0.049	−1.03
R cortex volume (mm^3^)	253,146 [28447]	275,597 [23308]	0.92	0.078	−0.9

There were no measurements with significant differences in the Benjamini-Hochberg critical values (q = 0.15) for 13 repeated t-tests. ISS, idiopathic short stature syndrome; SGA, short for gestational age; SD, standard deviation; L, left hemisphere; R, right hemisphere. GM, gray matter; WM, white matter; CSF, cerebrospinal fluid.

### 3.3 Voxel- and surface-based cortical analyses

**Figure 2:**
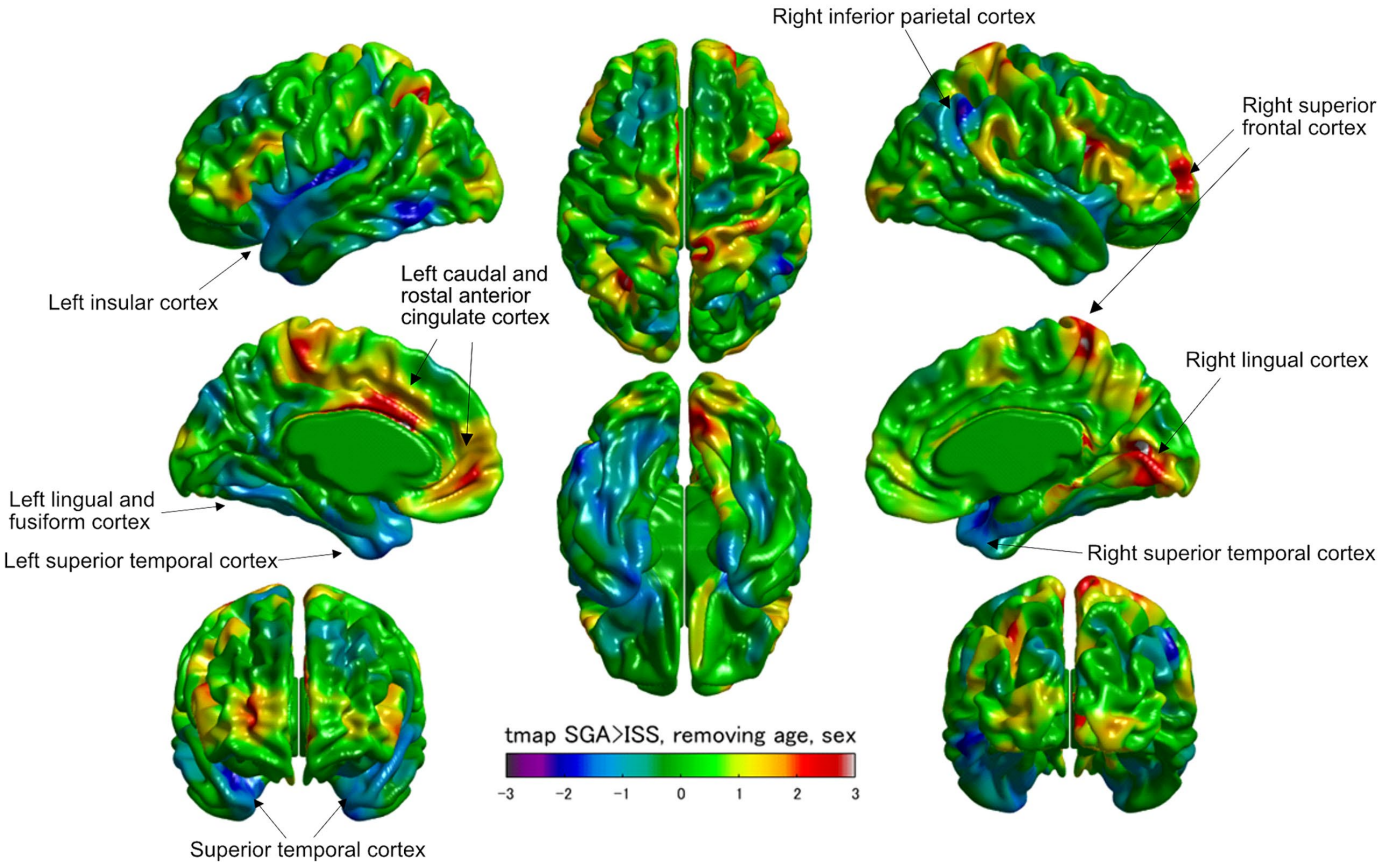
Visualized cortical thickness with a t-statistical map (tmap) demonstrating thicker or thinner lesions in patients born as SGA (SGA, *N* = 8) than those with ISS (ISS, *N* = 16). Blue and red color scales indicate less and greater mean cortical thicknesses in SGA, respectively.

Figure 2 illustrates a cortical thickness map superimposed on a 3D template brain surface image. The t-statistics with age and sex adjustments for the differences between the SGA and ISS groups demonstrated increased thickness in the left caudal and rostal anterior cingulate cortex, right lingual cortex, and right superior frontal cortex, and decreased thickness in the left insular cortex, left lingual and left fusiform cortex, right inferior parietal cortex, and bilateral superior temporal cortexes in SGA (Figure 2; Supplementary Table S1). Surface-based analyses using general linear multiple analysis (GLM) further revealed significant differences between the SGA and ISS groups. These differences were observed in the surface area of the left isthmus cingulate, left transverse temporal, left superior temporal, and right lingual gyri. Additionally, the left insular volume demonstrated significant differences between the two groups. Notably, no significant differences were observed in other areas, including thickness, volume, or GI of the cerebrum (Tables 3, 4; Supplementary Table S2).

**Table 3:** The candidate measurements using *t*-test in participants born as SGA without catch-up growth and those with ISS.

Category	Measurements (CIVET number)	SGA (*N* = 8) Mean [SD]	ISS (*N* = 16) Mean [SD]	The rate of SGA/ISS	*p*	Absolute Cohen’s *d*
Area	L Isthmus Cingulate Gyrus (19)	731 [62]	831 [66]	0.88	2.3 × 10^−3^	−1.55
Area	L Transverse temporal (28)	502 [45]	582 [55]	0.86	1.4 × 10^−3^	−1.54
Area	L Superior temporal (32)	4,372 [285]	5,076 [497]	0.86	2.4 × 10^−4^	−1.6
Area	R Lingual Gyrus (21)	2,510 [126]	2,879 [263]	0.87	1.2 × 10^−4^	−1.62
Volume	L Insula (7)	5,56 4[703]	6,609 [512]	0.84	3.3 × 10^−3^	−1.8
Volume	Lt Superior temporal (32)	14,013 [1476]	16,376 [1859]	0.86	3.5 × 10^−3^	−1.4
Volume	R Lingual Gyrus (21)	7,022 [295]	7,929 [702]	0.89	2.1 × 10^−4^	−1.5

Benjamini–Hochberg critical values (*q* = 0.15) for 241 repeating *t*-tests were determined as *p* < 4.4 × 10^−3^.

ISS, idiopathic short stature syndrome; SGA, short for gestational age; SD, standard deviation; L, left hemisphere; R, right hemisphere.

**Table 4:** The effects of covariates on candidate brain morphologic measurements; Univariate General Linear Model.

Category	Measurements	Adjusted R square	Corrected model	The presence of SGA	Age at scan	Sex
Area	L Isthmus Cingulate Gyrus (19)	0.404	***F* = 4.9** ***p* = 0.007**	***F* = 5.55** ***p* = 0.03**	*F* = 0.88 *p* = 0.36	*F* = 0.33 *p* = 0.57
Area	L Transverse temporal (28)	0.272	***F* = 3.15** ***p* = 0.038**	***F* = 6.01** ***p* = 0.024**	*F* = 0.36 *p* = 0.56	*F* = 0.14 *p* = 0.72
Area	L Superior temporal (32)	0.315	***F* = 3.64** ***p* = 0.023**	***F* = 8.61** ***p* = 0.0085**	*F* = 0.02 *p* = 0.89	*F* = 0.6 *p* = 0.45
Area	R Lingual Gyrus (21)	0.285	***F* = 3.29** ***p* = 0.033**	***F* = 10.1** **p = 0.005**	*F* = 0.4 *p* = 0.53	*F* = 0.055 *p* = 0.82
Volume	L Insula (7)	0.417	***F* = 5.11** ***p* = 0.0058**	***F* = 7.72** ***p* = 0.012**	*F* = 0.7 *p* = 0.41	*F* = 0.4 *p* = 0.53
Volume	L Superior temporal (32)	0.226	*F* = 2.68 *p* = 0.063	***F* = 7.27** ***p* = 0.014**	*F* = 0.52 *p* = 0.48	*F* = 0.012 *p* = 0.92
Volume	R Lingual Gyrus (21)	0.233	*F* = 2.74 *p* = 0.059	***F* = 7.13** ***p* = 0.015**	*F* = 0.006 *p* = 0.94	*F* = 0.0035 *p* = 0.95

Bold indicates statistically significant. ISS, idiopathic short stature syndrome; SGA, short for gestational age; SD, standard deviation; L, left hemisphere; R, right hemisphere.

## 4 Discussion

SGA births may stem from adverse conditions *in utero*, including insufficient nutrition and abnormal placental blood flow (Wu et al., 2004; Silver, 2018). Evidence indicates that SGA is associated with metabolic disorders, such as diabetes, and cardiovascular diseases in adulthood (Berkey et al., 1998; Barker et al., 2005; Mericq et al., 2017). Additionally, SGA births have been associated with neurological and psychological conditions such as ADHD (Heinonen et al., 2010). The absence of catch up growth is associated with subnormal intellectual and psychological performance (Lundgren et al., 2001) and aggressive behavior in later life (Takeuchi et al., 2018). Conversely, catch up growth demonstrates favorable effects on motor and language development, cognitive function, and academic achievement (Takeuchi et al., 2019).

Moreover, brain morphology is influenced by body height (Raz et al., 1997; Peters et al., 1998; Nopoulos et al., 2000; Posthuma et al., 2000; Koh et al., 2005; Heymsfield et al., 2009; Taki et al., 2012; Vuoksimaa et al., 2018). Children born as SGA who fail to achieve catch up growth by 3–4 years as defined by below −2 SD. SGA exhibited catch up growth until 2 years, but 10% demonstrated no catch up growth (Hokken-Koelega et al., 1995; Karlberg and Albertsson-Wikland, 1995). We used the data of previously reported healthy individuals (Shiohama et al., 2023) and observed differences between SGA individuals without catch-up growth or ISS and healthy individuals (Supplementary Table S3), confirming that body height influences brain morphology. Therefore, ISS was used as a control to exclude individuals with GHD or other abnormalities, thereby mitigating the impact of body size variability.

We analyzed surface and voxel measurements from structural brain MRI scans of patients born as SGA without catch up growth and ISS. Regional analyses and surface cortical maps by general multiple linear regression model, including age, revealed that areas of the left isthmus cingulate, left superior temporal, and right lingular gyri were significantly decreased in SGA without catch up growth compared with ISS. Additionally, the volume of the left insula was significantly lower in the SGA group without catch up growth compared to the ISS group. Furthermore, the t-statistics maps demonstrated a noteworthy decrease in thickness, specifically within the left insula in the SGA group without catch up growth compared to the ISS group. The left insula emerges as a focal point of distinction between the two groups, as both GLM and t-statistics analyses consistently revealed significant differences in this brain region. The left insula is crucial for regulating cognitive and emotional processes, with their development associated with size and interactions with other brain regions (Bush et al., 2000). The insula, which was previously associated with emotional and interceptive functions, is now recognized for its role in cognitive control and attention. Particularly, the anterior insula serves as a hub that facilitates interactions between brain networks that are involved in external attention and self-related cognition. It detects salient events, initiating control signals and facilitating access to attention and memory resources. It forms a “salience network,” together with the anterior cingulate cortex, indicating relevant stimuli to guide behavior. Basic mechanisms include bottom-up detection, network switching, autonomic modulation, and motor system access. This network model provides a simple explanation for the insula’s varied functions in typical adults and may elucidate affective and social–cognitive disorders (Menon and Uddin, 2010). Our findings reveal that prenatal environmental constraints induce alterations in brain structure, potentially contributing to neurological and psychological disparities SGA without achieving catch up growth although the occurrence of these changes in the uterine or later through other mechanisms remains unclear. SGA without catch up growth has more risk of subnormal intellectual and psychological performance even compared with those who achieved catch up growth (Lundgren et al., 2001). Our study assumes the association of the brain morphometry alteration in the insula of SGA without catch up growth with having more risk in intellectual and psychological performance. Regarding the mechanism causing morphometry change, our study excluded GHD with the GH stimulation test, so that morphological change in the brain was caused by different mechanisms. One possibility is an epigenetic factor. Recently, the risk of metabolic diseases and cardiovascular disease in infants born as SGA was explained by the epigenetic factor of DNA methylation which can be altered by environmental factors such as low nutrition or poor placental circulation (Thompson et al., 2010; Roifman et al., 2016; Hu et al., 2023). DNA methylation change alters protein expression, which results in the morphological brain change observed in this study. Future investigations are warranted to confirm the mechanism.

This study has several strengths. We accurately selected participants with ISS and SGA without achieving catch up growth using the GH stimulation test. This enabled us to assess the effect of catch up growth exclusion on brain morphology while controlling for height effects. Furthermore, we used a combination of analytical approaches and conducted a thorough morphometric analysis of brain structure in individuals with SGA without achieving catch up growth. However, this study has several limitations. First, the number of participants born as SGA without achieving catch up growth is limited, which is a relatively rare occurrence. Further analysis using a larger cohort of patients is warranted to validate our results. Second, causal effects cannot be inferred due to the cross-sectional nature of this study. Third, due to the small sample size after sex correction in brain morphometry analysis and the low frequency of SGA individuals without catch-up growth, a type II error may occur. Fourth, we used the CIVET pipeline due to its high reproducibility and familiarity with our research team, having previously established pediatric normal reference values using this tool (Shiohama et al., 2023). However, we acknowledge the need for confirmation with other pipelines, as the choice of pipeline may influence results. Finally, our findings provide valuable insights into the association between intellectual and psychological issues and SGA individuals without catch-up growth (Lundgren et al., 2001; Takeuchi et al., 2018), though specific cognitive tests or psychological scales were not conducted. Further prospective research is required to better understand the association of brain structure alterations in SGA individuals with psychological development. Despite its limitations, this study contributes to our understanding of the consequences of SGA without catch up growth on brain structure.

## 5 Conclusion

Patients born as SGA without catch up growth demonstrated specific morphological brain changes compared with ISS. Rather than GH, epigenetic change may be involved in the observed structural brain differences in patients born as SGA without catch up growth. The features of brain morphology in SGA without catch up growth determined in this study could contribute to further understanding of the association between SGA without catch up growth and both intellectual and psychological outcomes.

## Data Availability

The raw data supporting the conclusions of this article will be made available by the authors, without undue reservation.

## Footnotes

^1^ http://www.math.mcgill.ca/keith/surfstat/
