# Supplementary material for: Brain morphometric changes in children born as small for gestational age without catch up growth
Source: Front Neurosci. 2024 Aug 29;18:1441563. doi: 10.3389/fnins.2024.1441563 (PMC11390431; doi:10.3389/fnins.2024.1441563)
Supplement: Supplementary file 3 [file Table_3.DOCX]

| **Supplementary Table 3. The effects of covariates on candidate brain morphologic measurements;** **Univariate General Linear Model** | | | | | | |
| --- | --- | --- | --- | --- | --- | --- |
| Category | Measurements | Adjusted R square | Corrected model | The presence of ISS | Age at scan | Gender |
| Volume | Whole brain | 0.995 | **F = 2.00×10^3^**  **p < 0.001** | **F = 9.40**  **p = 0.004** | F = 2.80  p = 0.102 | **F = 18.85**  **p <0.001** |
| Volume | Cortical gray matter | 0.994 | **F = 1.54×10^3^**  **p < 0.001** | **F = 6.31**  **p = 0.016** | F = 4.07  p = 0.050 | **F = 18.69**  **p < 0.001** |
| Volume | White matter | 0.984 | **F = 0.58 ×10^3^**  **p < 0.001** | F = 3.67  p = 0.062 | **F = 12.31**  **p = 0.001** | F = 3.51  p = 0.068 |
| Volume | Subcortical gray matter | 0.995 | **F = 2.03 ×10^3^**  **p < 0.001** | **F = 8.59**  **p = 0.005** | F = 0.07  p = 0.791 | F = 7.12  p = 0.011 |

Bold indicates statistically significant. Abbreviation: ISS: idiopathic short stature syndrome
